# Deep Eutectic Solvent Assisted Dispersion of Carbon Nanotubes in Water

**DOI:** 10.3389/fchem.2020.00808

**Published:** 2020-09-17

**Authors:** Qammer Zaib, Idowu Adeyemi, David M. Warsinger, Inas M. AlNashef

**Affiliations:** ^1^Department of Civil and Environmental Engineering, University of Ulsan, Ulsan, South Korea; ^2^Department of Chemical Engineering, Khalifa University of Science and Technology, Abu Dhabi, United Arab Emirates; ^3^School of Mechanical Engineering and Birck Nanotechnology Center, Purdue University, West Lafayette, IN, United States; ^4^Department of Mechanical Engineering, Massachusetts Institute of Technology, Cambridge, MA, United States

**Keywords:** DES, carbon nanotubes, RSM, COSMO-RS, zeta potential, dynamic light scattering (DLS), polydispersity

## Abstract

Deep Eutectic Solvents (DESs) are emerging as a promising medium for many chemical processes. They can be used to observe specific properties required for nanomaterials' applications. Controlled CO_2_ adsorption requires disaggregation of carbon nanotubes into smaller bundles which can be accomplished by dispersing them in aqueous DES system. In this study, response surface methodology (RSM) was adopted to examine the impacts of three important factors on the dispersion of single walled carbon nanotubes (SWNTs) in Choline Chloride-Glycerol (ChCl-Gly) DES; (i) ChCl-Gly (mass% in water), (ii) sonication energy input (J/mL), and (iii) SWNTs' concentration (mg/L). The net negative surface charge of ChCl-Gly, a “green solvent,” provided superior dispersion of inherently negatively charged SWNTs in water via electrostatic repulsion. The impacts of the dispersion factors were quantified by the average aggregate diameter (nm) and polydispersity (polydispersity index, PDI) of SWNTs in aqueous-DES systems. Models were developed, experimentally verified, and statistically validated to map the impacts of these factors and to obtain optimized dispersions. The optimized dispersions, characterized by the small (<100 nm) and uniform (<0.1 PDI) SWNTs' aggregates, were achieved at lower sonication energy costs which can have promising implications across many nano-manufacturing fields. The dispersion/aggregation mechanism was proposed using COSMO-RS (based on equilibrium thermodynamics and quantum chemistry) modeling of ChCl-Gly and zeta potential measurements of SWNTs. This understanding will help create optimally sustainable and economically feasible DES-nanomaterial dispersions.

**Graphical Abstract:**
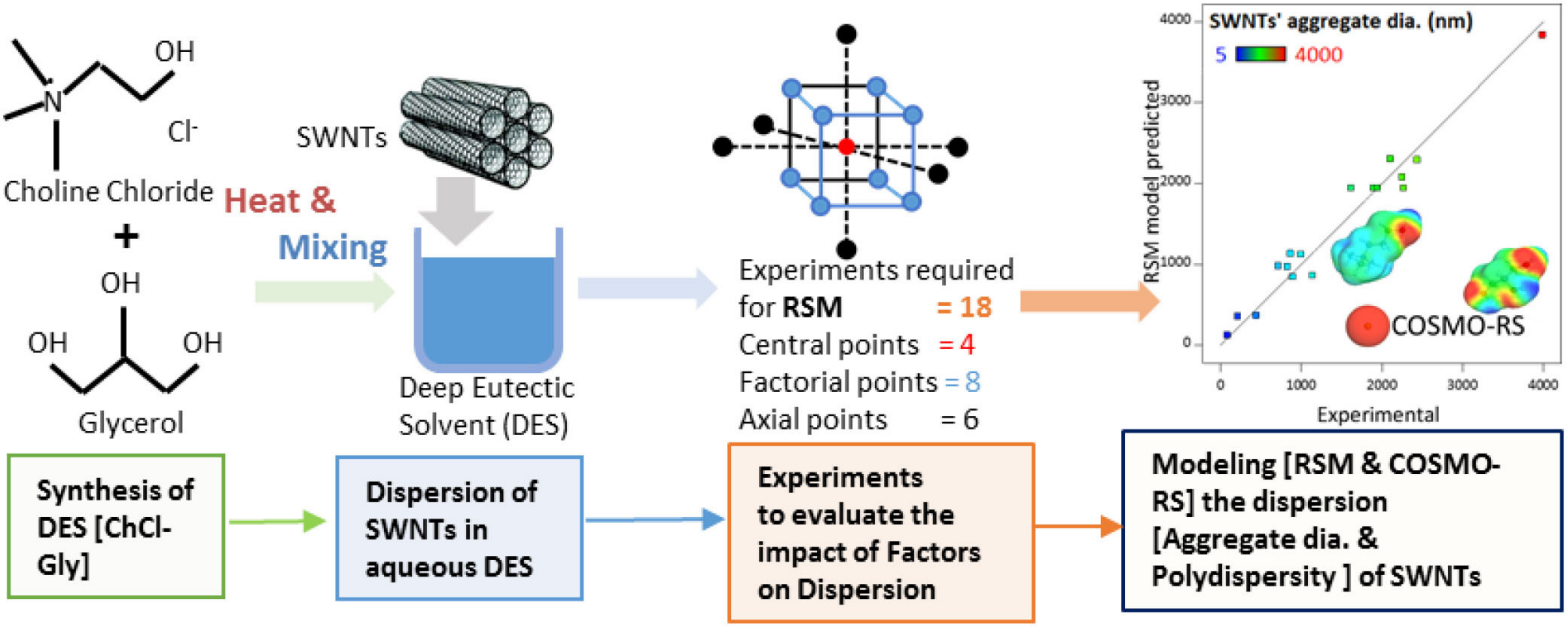
Experimental modeling to disperse SWNTs in aqueous DES.

## Introduction

Deep Eutectic Solvents (DESs) are mixtures of Brønsted or Lewis acids and bases that are emerging as a new class of solvents due to advantages in safety, simplicity, sustainability, cost, and applications that include synthesis and functionalization of nanomaterials (Abo-Hamad et al., 2015). DESs' have a variety of applications, including processing poorly soluble drugs (Morrison et al., 2009), biodiesel purification of glycerol (Abbott et al., 2007; Hayyan et al., 2010; Shahbaz et al., 2011; Zhang et al., 2012), organic synthesis (Ilgen and König, 2009), electrodeposition, metal processing (Smith et al., 2014), dye-sensitized solar cells, catalysis of polymers and fuel additives, and nanomaterial dispersion, functionalization, and fabrication (Zhang et al., 2012). They are being adopted as a “greener alternative” to conventional solvents because they are generally considered biodegradable, non-toxic, and safe (Smith et al., 2014). Though, DESs are generally considered environmentally benign, yet, only one of their four types (type III) could be considered as a truly “green” due to the absence of metal salts in their composition (Smith et al., 2014; Juneidi et al., 2015; Perna et al., 2020). Choline Chloride-Glycerol (ChCl-Gly) is one of the most popular type III DES. It is biodegradable and “practically harmless” to the aquatic ecosystem as reported in a study performed on fungi and fish (Juneidi et al., 2015). The LC_50_ dosage of aqueous ChCl-Gly for *Cyprinus carpio* fish was detected as high as >6,000 mg/L (Juneidi et al., 2015). Also, it was observed that aqueous ChCl-Gly is among the most readily biodegradable DESs. The 1 week and 28 days biodegradability of ChCl-Gly was recorded at 84 and 91%, respectively (Juneidi et al., 2015). Therefore, among the DESs, ChCl-Gly could be considered as a “green solvent.”

Carbon nanotubes (CNTs) have been known as a good candidate for CO_2_ adsorption owing to their high surface area, tunable pore structure, high ameanability to surface modification, and regerability for reuse (Wang et al., 2011; Rahimi et al., 2013). Some studies exhibited that CNTs carry superior adsorption capacity over silica materials and activated carbon despite same surface area (Cinke et al., 2003; Lu et al., 2008; Su et al., 2009; Lee et al., 2012). Cinke et al., for instance, observed one–fold higher adsorption capacity (at the cost of only 25% additional surface area) of single-walled carbon nanotubes (SWNTs) when compared with activated carbon (Cinke et al., 2003). They suggested that the larger pore sizes of SWNTs, as compared to other carbonaceous materials, allowed for easier adsorption of the CO_2_. The adsorption of CO_2_ on CNTs is reported to be governed by their intertube distance aka dispersion (Rahimi et al., 2013). Therefore, the adsorption of CO_2_ on SWNTs can be enhanced through dispersion. The dispersion of CNTs is often accomplished using hazardous chemicals (Datsyuk et al., 2008; Heister et al., 2010; Pramanik et al., 2017). We hypothise that the CNTs can be dispersed in water with the aid of ChCl-Gly DES, a green solvent. The improved surface area from dispersion in a ChCl-Gly would enhance the benefits that are inherent in CNTs for CO_2_ adsorption. To the best of our knowledge, the dispersion of single-walled carbon nanotubes (SWNTs) in the aqueous-DES system has not yet been systematically studied.

This work explores the effects of ChCl-Gly concentration in water, sonication energy, and SWNTs concentration (factors) on the dispersion of SWNTs (response) in the aqueous-DES system. The dispersion of SWNTs was evaluated by measuring their average aggregate diameter (nm) and polydispersity (polydispersity index, PDI), both of which were obtained by employing a dynamic light scattering technique (Krause et al., 2010; Masarudin et al., 2015). PDI is frequently used as an indicator for uniformity and stability of particles in suspensions; the lower the PDI values, the higher the number of evenly sized particles and vice versa. Together with aggregate diameter, PDI elaborates on the dispersion quality of the SWNTs in aqueous-DES systems. The experimental study was designed according to central composite rotatable design (CCRD), a statistical approach for using the response surface methodology (RSM). RSM is an efficient statistical technique for exploring the relationship between several explanatory (variables or) factors and (one or more) responses. The empirical models were developed which were statistically tested and validated before discerning the impacts of factors (ChCl-Gly concentration in water, sonication energy, and SWNTs concentration) on responses (average aggregate diameter and polydispersity of SWNTs). Moreover, the optimum experimental conditions were predicted to obtain desirable dispersions of SWNTs. The SWNTs' dispersion mechanism in aqueous DES system was proposed with the Conductor-like Screening Model for Realistic Solvents (COSMO-RS) for ChCl-Gly and zeta potential determination of SWNTs' aggregates. This study might be helpful in (i) understanding the dispersion of SWNTs in the aqueous ChCl-Gly DES system, (ii) obtaining optimum SWNTs dispersions in aqueous ChCl-Gly DES systems, (iii) estimating the fate and transport of SWNTs in aqueous DES environment, and (iv) designing similar studies with other nanomaterials and DESs.

## Materials and Methods

### Materials

Single-walled carbon nanotubes (SWNTs) were obtained from Sigma-Aldrich, synthesized by Catalytic Chemical Vapor Deposition Method (CoMoCAT® CVD). They were over 95% pure with an internal diameter of 0.6–1.1 nm and a bulk density of 0.128 g/cm3 according to the manufacturer. Choline Chloride and Glycerol (99 wt.%) were acquired from Acros chemicals (Belgium). All the materials were used as received, without further treatment, to mimic regular laboratory and industrial practices. Milli-Q water, having a resistivity ≥18.2 MΩ.cm at 25°C, was used in all experiments.

### Synthesis of DES

The synthesis method utilized for the ChCl-Gly DES in this study is based on the approach suggested by Abbott and co-workers (Abbott et al., 2003). In this approach, an adequate amount (1:2 molar ratio of ChCl:Gly) of the Bronsted or Lewis acid and base were mixed in a well-sealed vial. The mixture of these acid and base was then thoroughly shaken at 90°C until a homogeneous liquid was observed. The resulting DES was left to cool down to room temperature.

### SWNTs' Dispersions in DES

SWNTs' dispersions in aqueous-DES systems were created by adding the predetermined (according to experimental design) mass of SWNTs in a pre-mixed water-ChCl-Gly mixture followed by sonication. Sonication was performed to disperse the SWNTs' agglomerates. The sonication energy was pre-calibrated using NIST protocol and following the previously reported method, details of which can be found elsewhere (Taurozzi et al., 2012; Zaib and Ahmad, 2020). The dispersed SWNTs were then centrifuged at ~18,000 g-force using Eppendorf™ 5810R centrifuge. Thereafter, samples from the middle portion of the suspension were drawn for characterization.

### Characterization of SWNTs' Dispersions

SWNTs' dispersions were characterized by measuring their aggregate size and uniformity by employing dynamic light scattering (DLS) using a ZetaPALS particle analyzer, a product of Brookhaven Instruments Corp. (Holtsville, N.Y.). The instrument measures the effective diameter of a nanoparticle in a liquid environment by calculating its hydrodynamic diameter (i.e., the size of a sphere that has the same diffusion behavior as that of the measured particle). Although it is impossible to estimate real size and shape of carbon nanotubes via DLS and the technique is only capable of estimating the degree of dispersion, still, it is one of the most frequently used methods in estimating bundle sizes of SWNTs (Murdock et al., 2008; Zaib et al., 2012; Khan et al., 2013; Ma and Larsen, 2013; Zaib and Ahmad, 2019). The polydispersity describes the degree of “non-uniformity” of a distribution. It can be expressed in terms of the polydispersity index (PDI). The value of PDI ranges from 0 (absolutely monodispersed) to 1 (perfectly polydispersed) (Murdock et al., 2008; Ma and Larsen, 2013). In general, the PDI ≤ 0.1, 0.1–0.4, and ≥0.4 represent highly monodispersed, moderately monodispersed, and polydispersed colloidal dispersions (Bhattacharjee, 2016). PDI can be mathematically represented (Murdock et al., 2008; Clayton et al., 2016) as:

$$\begin{array}{l} PDI=\;{\left(\frac{\sigma \;}{d\;}\right)}^{2}=\;{\left(\frac{Standard\;deviation\;}{Mean\;diameter\;of\;particles\;}\right)}^{2} \end{array}$$

### Statistical Experimental Design, Analysis, and Model Fitting

**Figure 1:**
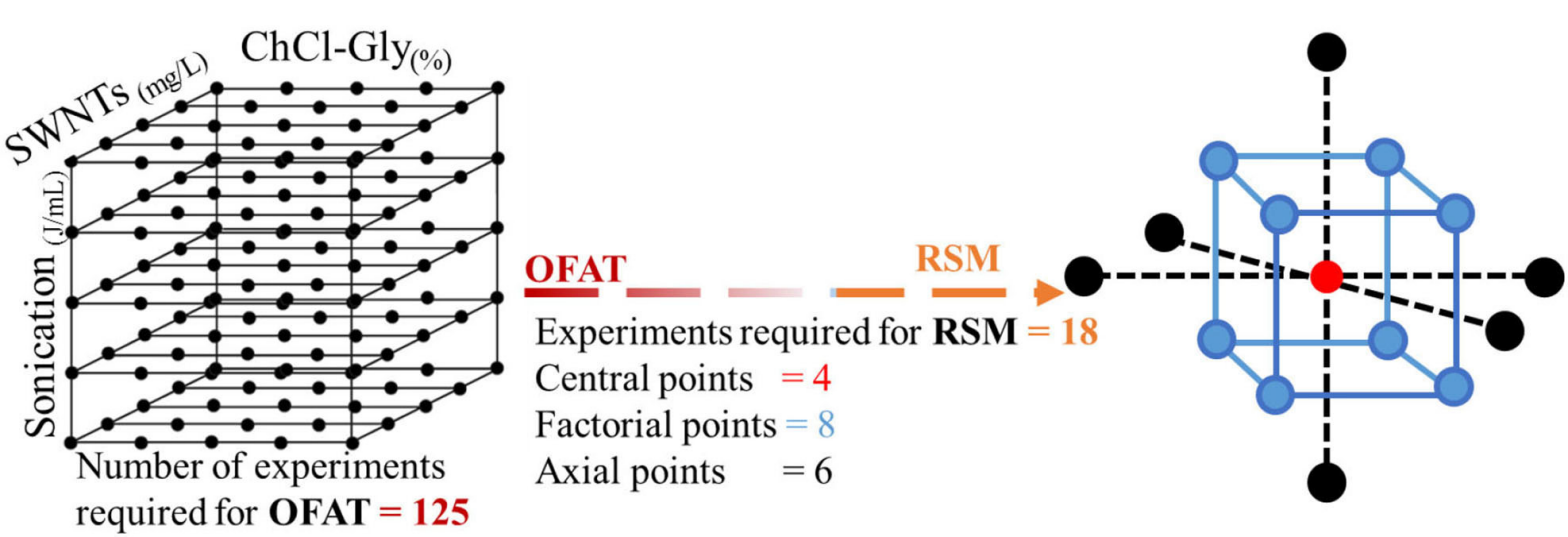
(LHS) One factor at a time (OFAT) for three variables [A: ChCl-Gly (mass% in water), B: SWNTs' concentration (mg/L), and C: Sonication energy (J/mL)] at five levels require 53 (=125) experiments without replicates. (RHS) Response surface methodology (RSM) reduces the number of experiments to 18 only. The circular points (at center, corners, and parallel to the face of the cube) represent the experimental runs. Central composite rotatable design (CCRD) of RSM, representing experimental points in the design space for three variables, is capable of navigating through the design space which is not possible with OFAT.

Central composite rotatable design (CCRD) of experiments was selected for this study. CCRD reduced the number of experiments by 86%, introduced variability (four experimental runs at center), and enabled navigation throughout the entire experimental space, as shown in Figure 1. Design Expert software (Stat-Ease Inc., Minneapolis, MN) was used for the statistical design of experiments. Three independent variables (factors), namely Choline Chloride-Glycerol (mass% in water), SWNTs concentration (mg/L), and Sonication (J/mL) were studied for aggregate diameter (nm) and polydispersity (PDI) of SWNTs. The empirical models representing the impacts of factors (ChCl-Gly mass percent, sonication energy, and SWNTs' conc.) on responses (aggregate diameter and polydispersity of SWNTs) were developed using response surface methodology (RSM) (Anderson and Whitcomb, 2016; Myers et al., 2016). Choline chloride-Glycerol mass percent in water was varied from 0 to 100% where ChCl:Gly 0 percent implies the complete absence of ChCl:Gly and ChCl:Gly 100% represents absolute ChCl-Gly DES. Therefore, this study encompasses the entire range of possibilities for processing the SWNTs' dispersions in water only, ChCl:Gly only, and their mixtures. Each variable was studied at five levels represented by –α, −1, 0, 1, and α as shown in Table 1. A total of 18 different combinations were prepared in random order according to the CCRD configuration. The experimental runs describing the combination of factors and their experimental responses are shown in Table 2. The SWNTs' dispersions, obtained from suggested experimental runs, were characterized by the techniques mentioned in the “Characterization of SWNTs' Dispersions” section.

**Table 1:** Experimental factors and their levels studied in central composite rotatable design (CCRD) of experiments.

	**Factors**	**Units**	**Levels**				
			**–α**	**−1**	**0**	**+1**	**+α**
A	ChCl-Gly [DES]	%	0	20	50	80	100
B	SWNTs conc.	mg/L	1	21	51	80	100
C	Sonication	J/mL	20	36	60	84	100

*Each experimental factor was studied at five levels (–α, −1, 0, 1, α). Coded levels of experimental factors and their corresponding actual values are shown above*.

**Table 2:** Experimental design matrix of the three variables in the design space of central composite rotatable design (CCRD) of experiments.

	**Factors**			**Responses**			
**Exp**.	**ChCl-Gly [DES]**	**SWNTs conc**.	**Sonication**	**SWNTs' aggregate diameter**		**Polydispersity**	
	**(%)**	**(mg/L)**	**(J/mL)**	**(nm)**		**(PDI)**	
				**Exp**.	**Pred**.	**Exp**.	**Pred**.
1	50	51	20	3,989	3,836	0.88	0.85
2	50	1	60	894	846	0.22	0.19
3	20	80	84	442	367	0.13	0.14
4	20	21	84	213	357	0.11	0.13
5	50	51	60	2,268	1,944	0.38	0.36
6	80	80	36	869	1,133	0.25	0.28
7	0	51	60	86	124	0.09	0.09
8	20	21	36	2,437	2,293	0.55	0.52
9	50	51	100	2,251	2,080	0.5	0.46
10	100	51	60	17	0	0.02	0
11	50	51	60	1,618	1,944	0.3	0.36
12	80	21	84	830	970	0.15	0.20
13	50	51	60	1,895	1,944	0.37	0.36
14	20	80	36	2,106	2,303	0.48	0.53
15	80	80	84	716	980	0.18	0.21
16	50	51	60	1,941	1,944	0.38	0.36
17	50	100	60	1,140	863	0.26	0.21
18	80	21	36	998	1,123	0.22	0.27

The data obtained were then fitted to polynomial equations to model (empirically) the relationship between factors and responses. Statistical analyses of regression models representing SWNTs' dispersions (aggregate diameter and polydispersity) in aqueous DES systems were performed. The significance of models was evaluated by analysis of variance (ANOVA). The less significant coefficients were eliminated after the F-test to obtain the final model. The diagnostics were then performed on the models before using them to predict the aggregate diameter and polydispersity of SWNTs in aqueous-DES systems with various mass percent of ChCl-Gly DES.

### Molecular Modeling and Zeta Potential

The conductor-like screening model for a real solvent (COSMO-RS) was adopted to estimate the surface charge of ChCl-Gly DES. COSMO-RS quantifies the interaction energy of ChCl-Gly DES's interacting species through polarization charge densities. The computations were performed using COSMOthermX software package by first generating the optimized geometry of ChCl-Gly species in TURBOMOLE (graphical user interface TmoleX). The Zeta potentials of SWNTs were measured using ZetaPALS particle analyzer described above. The instrument employs phase analysis light scattering to measure the electrophoretic motilities of SWNTs' aggregates. The Smoluchowski equation was used to calculate zeta potentials from electrophoretic mobilities (Hu et al., 2005).

## Results and Discussion

### DES-Water Mixture

Although sustaining a deep eutectic solvent in aqueous media is challenging, some studies have carefully investigated the DESs-water mixtures (Abbott et al., 2003, 2014; Shah and Mjalli, 2014; Dai et al., 2015; Hammond et al., 2017; Abdel Jabbar and Mjalli, 2019). Choline chloride-glycerol and some other DESs exhibited a good capacity for holding significant amounts of water whilst maintaining their eutectic characteristics (Abbott et al., 2003, 2014). Hammond and co-workers observed that the nanostructure of a deep eutectic solvent (reline: choline chloride/urea/water) was maintained in the presence of a remarkably high quantity of water (~40 wt.%) (Hammond et al., 2017). They explained that this retention of their eutectic nature was due to the solvophobic sequestration of water into nanostructured domains around cholinium cations. In an attempt by Dai et al. (2015) to decrease the viscosity of some DESs, it was demonstrated (using FTIR and NMR studies) that a strong hydrogen bonding between the two components of the DES was present even after adding a significant amount of water (~25% v/v). Mjalli et al. reported that the thermo-physical properties and ultrasonic behavior of some DESs were sustained under significant water addition (Shah and Mjalli, 2014; Abdel Jabbar and Mjalli, 2019). They have hypothesized that in the presence of water, the behavior of the DESs were retained because the anion was preferentially hydrated as compared to the cholinium cation and urea. This effect is similar to what was reported by Hammond et al. (2017).

### Model Development and Analysis

Second-order polynomial equations were developed to fit the experimental data of SWNTs aggregate diameter and polydispersity. The input variables to these equations were: A: ChCl-Gly (mass% in water), B: SWNTs' concentration (mg/L), and C: Sonication energy (J/mL). The variable selection technique was used to find good fits between these parameters, and yield statistically acceptable regression coefficients. Stepwise regression was performed on a quadratic model to represent SWNTs' aggregate diameter (Equations 1, 2) and polydispersity (Equations 3, 4) in aqueous ChCl-Gly system. Equations 2, 4 are essentially the same as Equations 1, 3, respectively, except that they are corrected for units. Consequently, Equations 1, 3 (coded) can be helpful for estimating the comparative impact of variables whereas Equations 2, 4 could be used to estimate the aggregate diameter and polydispersity of SWNTs in our aqueous ChCl-Gly system (and similar aqueous-DES systems). Empirical models representing SWNTs' aggregate diameter and polydispersity in the aqueous DES system are presented in Table 3.

**Table 3:** Empirical models representing SWNTs aggregate diameter and polydispersity in the aqueous ChCl-Gly DES system.

**Parameter**	**Model**	**Coded/ Actual**	**Eq**.
SWNTs' aggregate diameter (nm) =	1944.4**–**139.2A+5.0B**–**522.2C+445.9AC**–**726.6A^2^**–**385.2B^2^+358.3C^2^	Coded	1
	4470.4+39.7 ChCl-Gly. [DES]+45.1 SWNTs' conc.**–**129.5 Sonication+0.6 ChCl-Gly. [DES]*Sonication**–**0.8 ChCl-Gly. [DES]^2^**–**0.4 SWNTs' conc.^2^+0.6 Sonication^2^	Actual	2
SWNTs' Polydispersity index (PDI) =	0.36**–**0.04A+0.006B**–**0.11C+0.08AC**–**0.12A^2^**–**0.06B^2^+0.1C^2^	Coded	3
	1.2+0.005 ChCl-Gly. [DES]+0.007 SWNTs' conc.**–**0.03 Sonication+0.0001 ChCl-Gly. [DES]*Sonication**–**0.0001 ChCl-Gly. [DES]^2^**–**0.0001 SWNTs' conc.^2^+0.0002 Sonication^2^	Actual	4

**Figure 2:**
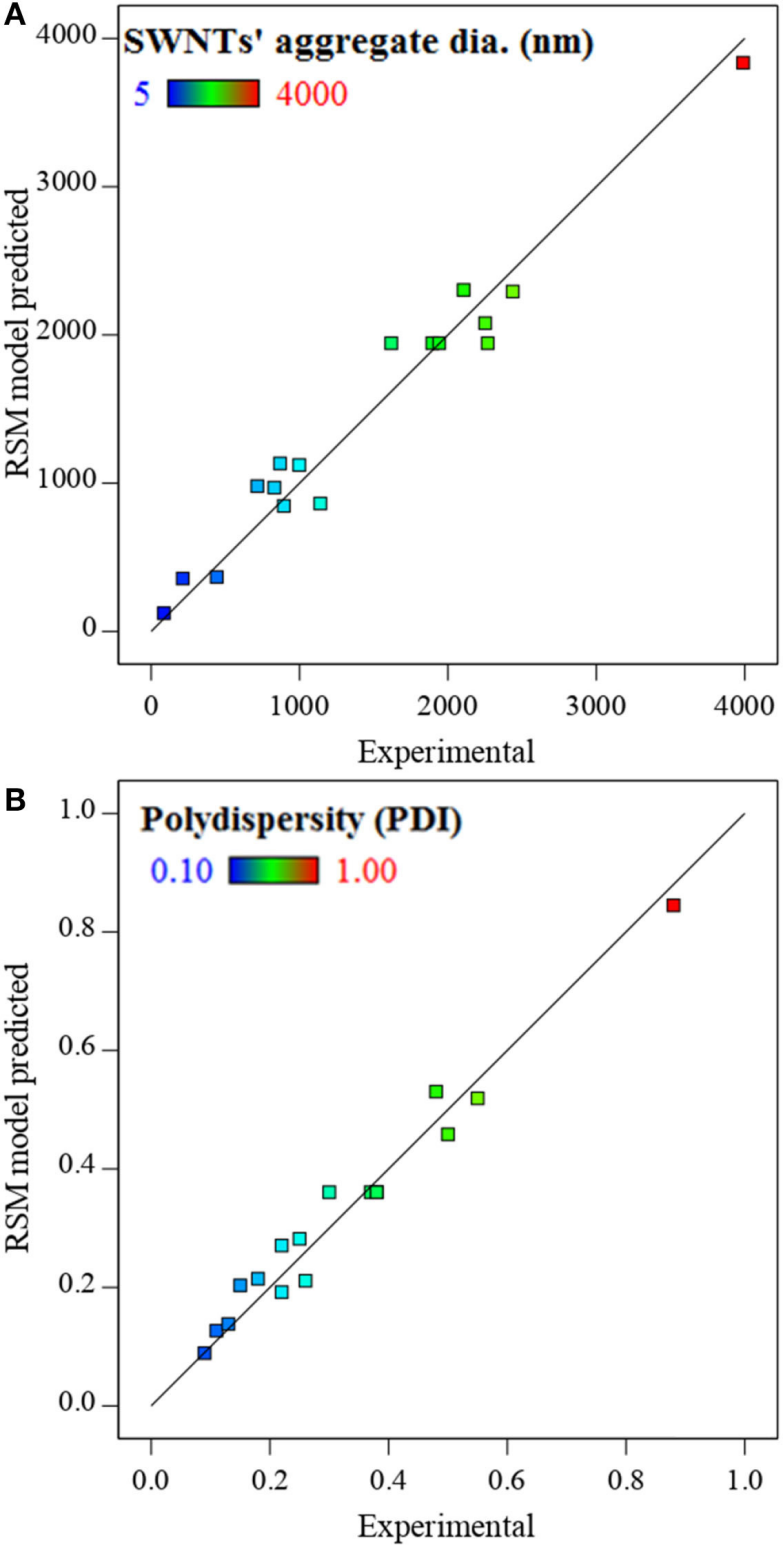
RSM model predicted vs. experimentally observed values of SWNTs' **(A)** aggregate diameter and **(B)** polydispersity. The straight line (y = x) is a reference for perfect prediction.

The predictabilities of the models for SWNTs aggregate diameter and polydispersity are shown in Figure 2. The straight lines, in the figures, represent the perfect prediction. The experimental vs. model predicted values are closely distributed around the perfect prediction line (y = x). The regression coefficient (*R*^2^) values for SWNTs' aggregate diameter and polydispersity were consistently above 0.95. This high correlation suggests a good agreement between experimental and model-predicted values and recommends the suitability of the models to represent the experimental data. The models were statistically evaluated through ANOVA.

### Statistical Evaluation of the Models

There are several ways to check the adequacy of the models (Montgomery, 2008; Myers et al., 2016). The statistical significance of the models and their terms can be evaluated by ANOVA, in which higher *F*-value corresponds to higher significance in the model fitting the data. To ensure the adequacy of the models, the following statistical analyses were performed: *F*-tests, the lack of fits tests, calculation of the coefficient of determination, and estimation of adequate precision (signal/noise) values.

The ANOVA results of the models are shown in Table 4. Firstly, the *F*-values of 33.1 (for aggregate diameter) and 36.0 (for polydispersity) imply that the models are significant. Secondly, the F-values for the lack of fits (relative to pure error) for both models were insignificant (i.e., 1.06 for Equation 1 and 2.24 for Equation 3). Thus, for these two equations, there are a 53.1 and 27.2% chances that these high lack of fit F-values occur due to noise for the model developed to represent SWNTs' aggregate diameter (Equation 1) and polydispersity (Equation 3), respectively. Additionally, the statistical significance of the models was confirmed by their coefficient of determination (*R*^2^), adjusted *R*^2^, and predicted *R*^2^. The adjusted *R*^2^, unlike *R*^2^, only increases when the relevant variables are added to the model (Anderson and Whitcomb, 2016). The values of *R*^2^ and adjusted *R*^2^ are ≥0.93 indicating the robustness of the models. The predicted *R*^2^ values of 0.84 (for both models) are quite high and in reasonable agreement with adjusted *R*^2^ showing the good predictability of the models (Anderson and Whitcomb, 2016; Myers et al., 2016). Also, the adequate precision values (a measure of signal to noise) are 23.08 and 25.59 for the two models, which are significantly higher than the desired value of 4 (Anderson and Whitcomb, 2016). Therefore, both models were considered suitable to navigate through the experimental design space.

**Table 4:** ANOVA of models developed for SWNTs aggregate diameter and polydispersity in the aqueous ChCl-Gly DES system.

**Source**	**Sum of Squares**	**df**	**Mean Square**	***F*-value**	***p*-value**	**Significance**
**SWNTs' aggregate dia**.						
Model	1711,0000	7	244,5000	33.11	<0.0001	Significant
A-ChCl-Gly. [DES]	26,4600	1	26,4600	3.58	0.0876	
B-SWNTs' conc.	346	1	346	0.0047	0.9468	
C-Sonication	372,4000	1	372,4000	50.43	<0.0001	
AC	159,0000	1	159,0000	21.54	0.0009	
A^2^	667,7000	1	667,7000	90.42	<0.0001	
B^2^	187,7000	1	187,7000	25.41	0.0005	
C^2^	162,4000	1	162,4000	21.99	0.0009	
Residual	738,500	10	73,849			
Lack of Fit	52,5600	7	75,080	1.06	0.5313	Not significant
Pure Error	21,2900	3	70,978			
R^2^	0.9568		Adj. R^2^	0.9297		
Pred. R^2^	0.8395		Adeq. Precision		23.08	
**Polydispersity**						
Model	0.702	7	0.10	36.0	<0.0001	Significant
A-ChCl-Gly. [DES]	0.025	1	0.03	9.08	0.0131	
B-SWNTs' conc.	0.000	1	0.00	0.16	0.7003	
C-Sonication	0.180	1	0.18	64.69	<0.0001	
AC	0.053	1	0.05	18.95	0.0014	
A^2^	0.187	1	0.19	67.16	<0.0001	
B^2^	0.040	1	0.04	14.35	0.0036	
C^2^	0.134	1	0.13	48.04	<0.0001	
Residual	0.028	10	0.00			
Lack of Fit	0.023	7	0.00	2.24	0.2725	Not significant
Pure Error	0.005	3	0.00			
R^2^	0.9618		Adj. R^2^	0.9315		
Pred. R^2^	0.8371		Adeq. Precision		25.59	

**Figure 3:**
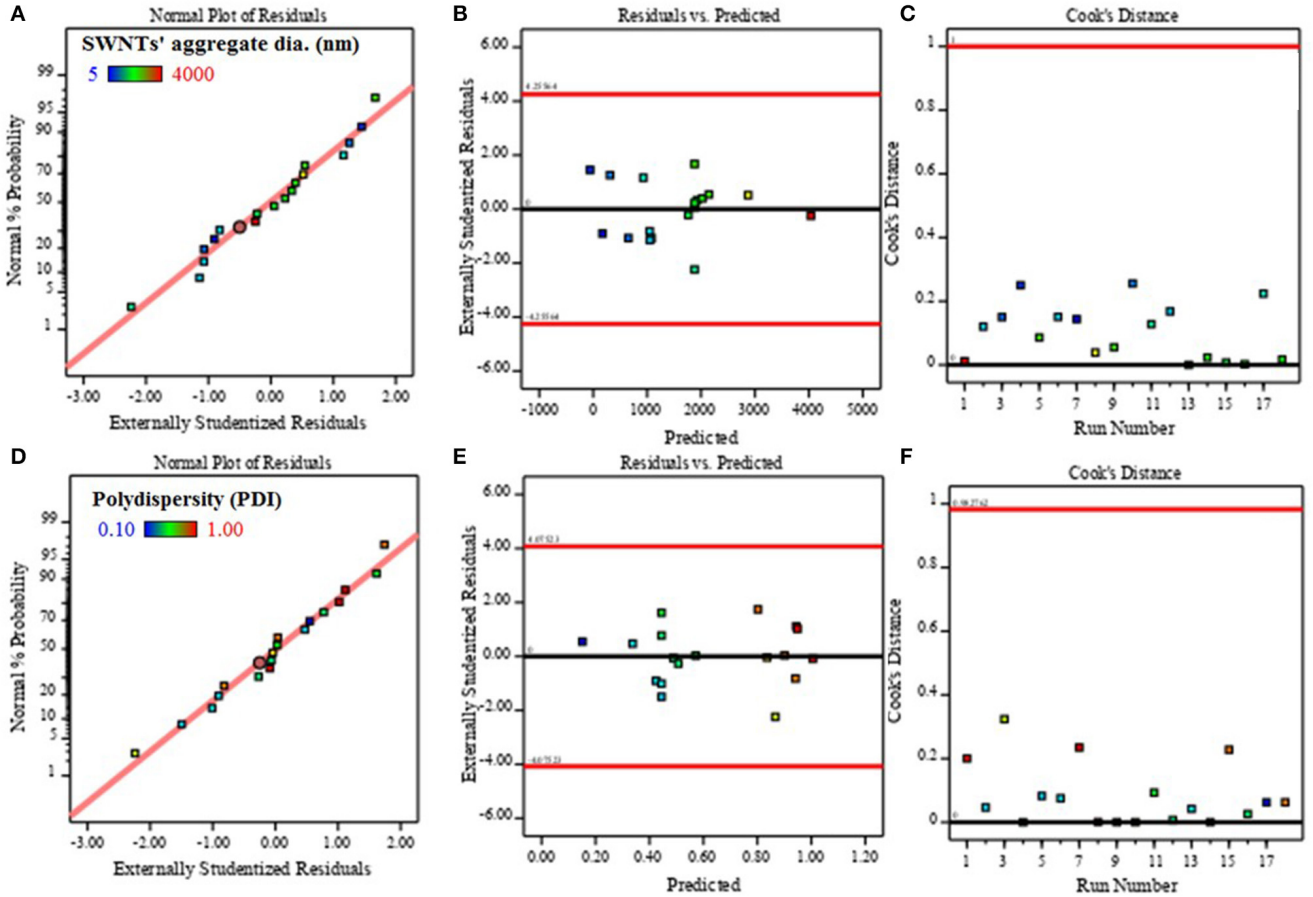
The **(A)** normal distribution of residuals, **(B)** absence of pattern in residuals vs. predicted response, and **(C)** non-existence of outliers in cook's distance plots for SWNTs' aggregate diameter and **(D,E,F)** polydispersity.

Statistical diagnostics were performed to validate the adequacy of the models. The results of the diagnostics for SWNTs' aggregate diameter are presented in Figures 3A–C and that of polydispersity are shown in Figures 3D–F i.e., the figure shows the normal plots of residuals (A,D), residuals vs. predicted plots (B, E), and cook's distances (C, F). Figures 3A,D revealed that the residuals generally fall on a straight line implying that errors are distributed normally, and thus, support adequacy of the least-square fit. Furthermore, Figures 3B,E affirmed the absence of obvious pattern and unusual structure. They exhibited uniform scatter above and below the x-axis, which implies that the proposed models are adequate and there is no reason to suspect any violation of the independence or constant variance assumption. Cook's distance is plotted in Figures 3C,F. There, too, none of the experimental point (run number) lies above the red line exhibiting the absence of outliers. The good correlation between experimental vs. model predicted values (Figure 2), ANOVA (Table 4), and diagnostics (Figure 3) suggest the statistical suitability of the models (Table 3) to represent SWNTs' aggregate diameter and polydispersity in aqueous DES systems. Therefore, the models were used to evaluate the impact of factors on SWNTs dispersion in the aqueous ChCl-Gly system.

### Impact of Primary Factors on the Dispersion of SWNTs

**Figure 4:**
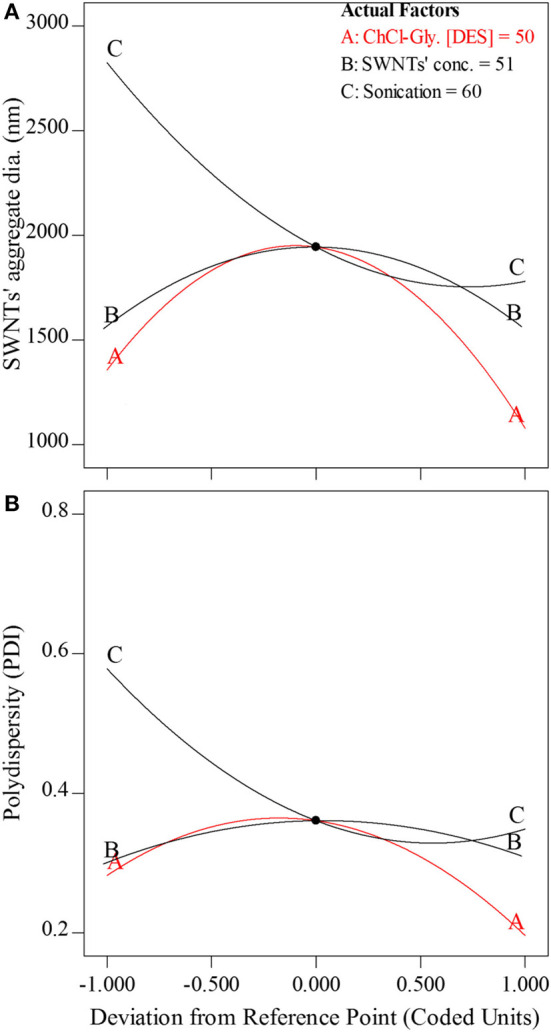
Modeled perturbation plots showing the effect of one variable at a time (while keeping the other two constant) on **(A)** SWNTs' aggregate diameter (nm) and **(B)** polydispersity (PDI) in aqueous ChCl-Gly system. The coded units on the x-axis can be inferred from the experimental values shown in Table 1. The coded value of 0.0 corresponds to 50% mass of ChCl-Gly, 51 mg/L SWNTs' concentration, and 60 J/mL sonication energy input to the aqueous DES system.

The perturbation plots show the comparative effects of individual variables, one at a time, on SWNTs' aggregate diameter (Figure 4A) and polydispersity (Figure 4B). The response of each variable [A: ChCl-Gly (mass% in water), B: SWNTs' concentration (mg/L), and C: Sonication energy (J/mL)] was recorded while keeping the other two at their respective middle levels as shown in perturbation plots (Figure 4). Figure 4A shows the variability in SWNTs' aggregate diameter as a function of individual input parameters. The SWNTs' aggregate diameter increased upon increasing the concentration of ChCl-Gly in aqueous DES solution up to a certain limit (20–50% ChCl-Gly) and then decreased (50–80%). This observation shows that SWNTs tend to aggregate in aqueous-DES solution and the aggregation is highest at ~50% DES in water.

This study and past studies have given insight into the impact of charge screening and hydrogen bonding on SWNT aggregation. Dai et al. (2015) observed the weakening of H-bonding interactions between the two components of some natural DESs upon the addition of water. In that study, the H-bonding completely disappeared as the water concentration reached ~50–80% in Choline Chloride based DESs. Therefore, it can be assumed that the highest aggregation of SWNTs at ~50% aqueous-DES solution could be due to the absence of ChCl-Gly DES at that experimental conditions. Instead, the ChCl-Gly dissociated into its constituents and increased the ionic conductivity of the water (Dai et al., 2015). The increase in ionic conductivity of water is frequently reported to further SWNTs' aggregation by screening electrostatic charge and thereby suppressing electrostatic repulsion between negatively charged SWNTs (Saleh et al., 2010). In the present study, the SWNTs' aggregate diameter increased from 1,378 to 1,963 nm with the increase in DES concentration from 20 to 50% and then decreased to 1,086 nm as the DES concentration approached 80%. The impact of SWNTs' concentration on its aggregation followed a similar trend to that of DES concentration. However, comparing the slopes of the two variables, the impact of DES concentration appears to be more pronounced than that of SWNTs' concentrations (Anderson and Whitcomb, 2016). Here, too, the biggest aggregates of SWNTs were observed at a 50% aqueous-DES solution.

The impact of sonication energy on SWNTs' aggregate diameter was largely linear with a positive slope (from ~36 J/mL to ~70 J/mL) indicating their nearly direct relationship (Anderson and Whitcomb, 2016). The sonication energy input, in general, decreased SWNTs' aggregate diameter, which is a commonly observed phenomenon. It is why the sonication is performed for dispersing carbon nanotubes in the first place (Di Crescenzo et al., 2009; Saleh et al., 2010; Zaib et al., 2012; Zaib and Ahmad, 2019). The further increase in sonication energy could not significantly reduce SWNTs' aggregate diameter as observed earlier in ionic liquid-based surfactant (Di Crescenzo et al., 2009) and water (Koh et al., 2011; Zaib et al., 2012) backgrounds. Figure 4B shows the effects of factors on polydispersity. The effects of factors on polydispersity are similar to that of SWNTs' aggregate diameter (Figure 4A). The polydispersity decreased with the decrease of SWNTs' aggregate diameter and vice versa. Therefore, it can be inferred that the small SWNTs aggregates with relatively low polydispersity could be obtained at either low (0–20%) or high (80–100%) ChCl-Gly concentrations and sonication energy input ≥70 J/mL.

### Combined Effects of Factors (ChCl-Gly Percent in Water, Sonication Energy, and SWNTs Concentration) on the Dispersion of SWNTs

**Figure 5:**
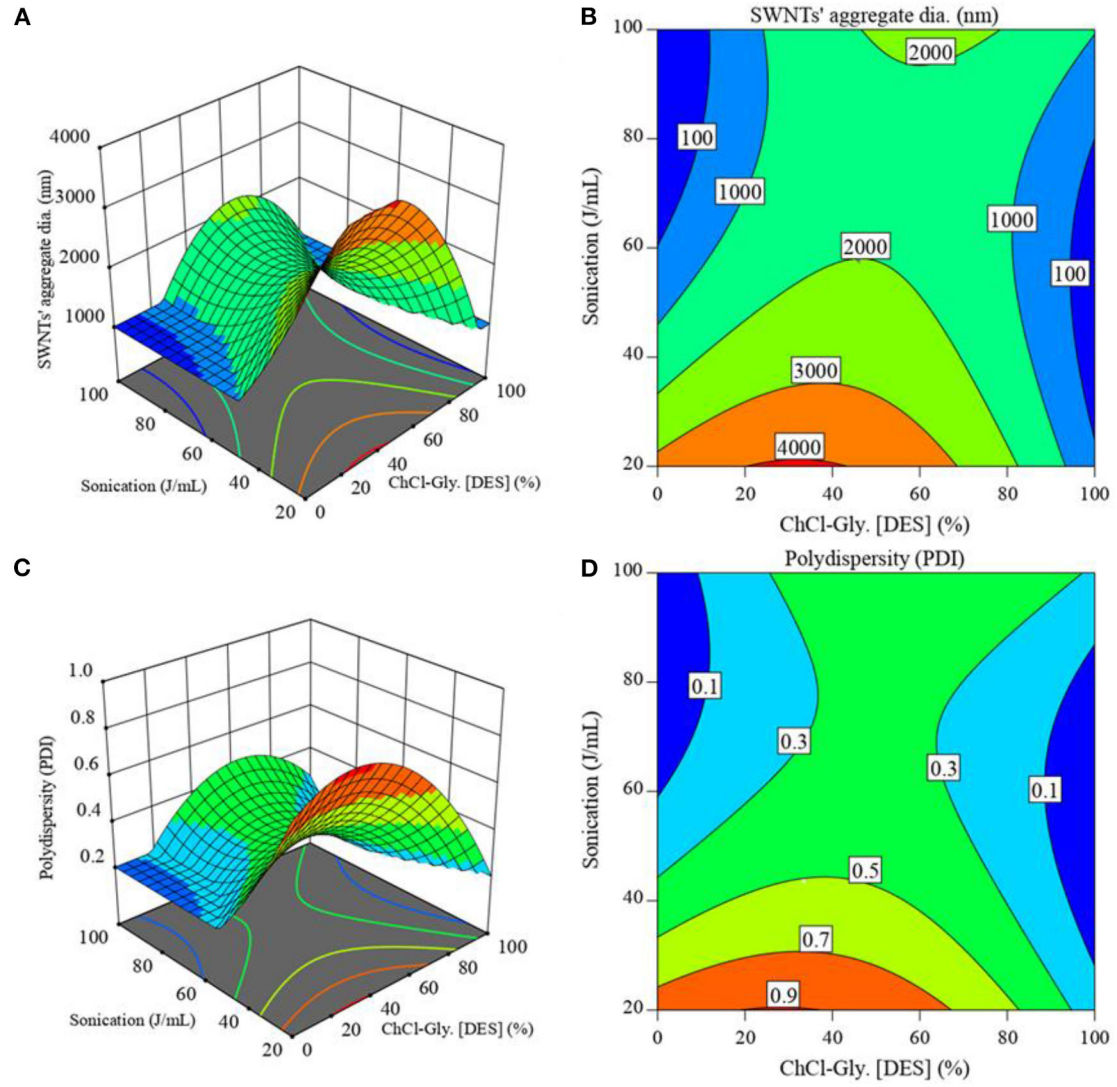
Combined impacts of ChCl-Gly concentration (mass%) and sonication (J/mL) on **(A,B)** SWNTs' aggregate diameter (nm) and **(C,D)** polydispersity (PDI). The SWNTs' concentration was fixed at 50 mg/L. Conditions for superior dispersion of SWNTs are indicated by low values of aggregate diameter and PDI approaching 0 (both being blue). Ideal performance occurs at very low and high concentrations of the DES, and notably, the DES can achieve high performance at lower sonication energies than the usual solvent, water.

Figure 5 shows the response surface plots and their corresponding contour plots of SWNTs' aggregates diameter (Figures 5A,B) and polydispersity (Figures 5C,D) vs. the significant pair of factors from ANOVA, Table 4 (i.e., AB: ChCl-Gly^*^Sonication). These plots can help identify the regions where desired SWNTs' aggregate diameter and polydispersity are most probable. The interactions between the variables (ChCl-Gly and Sonication) are evident from the curvatures in the figure. Figures 5A,B shows the contours of varying aggregate diameter resulting from combined interactions of ChCl-Gly concentrations (%) and sonication energy inputs (J/mL). The biggest SWNTs' aggregates (≥4,000 nm) can be expected at 20–43% DES concentration and ≤22 J/mL sonication energy input. Noticeably smaller SWNTs' aggregates (≤1,000 nm) occur at either low (≤20%) or high (≥80%) DES concentrations represented by blue bands in the respective plots. This observation agrees with the findings from the perturbation plot (Figure 4A) which predicted small SWNTs aggregates either at very low or very high DES concentrations. However, at low ChCh-Gly concentration (≤20%) higher sonication energy (>45 J/mL) is required whereas at high ChCh-Gly concentration lower sonication energy (≥20 J/mL) results in similar dispersion quality. This might be due to the increase in viscosity and boiling point of the aqueous ChCl-Gly systems at higher concentrations of ChCh-Gly DES (Dai et al., 2015). The high viscosity and boiling point of the receiving liquid delays the growth of sonication cavities (time from formation to implosion) and ultimately results in effective utilization of sonication energy (Santos et al., 2009). The combined effect of ChCl-Gly concentration and sonication energy input on polydispersity follows a similar trend as shown in Figures 5C,D. There too, uniform dispersions (PDI < 0.1) were obtained either at low ChCl-Gly concentrations (≤12%) combined with high sonication (≥60 J/mL) or high ChCl-Gly concentrations (≤90%) at medium sonication (30–85 J/mL). Figure 5 can be helpful in identifying the combination of factors (ChCl-Gly conc., SWNTs' conc. and sonication energy) to obtain desirable SWNTs' dispersions with appropriate aggregate diameter and suitable polydispersity for an intended application.

### Optimization

**Figure 6:**
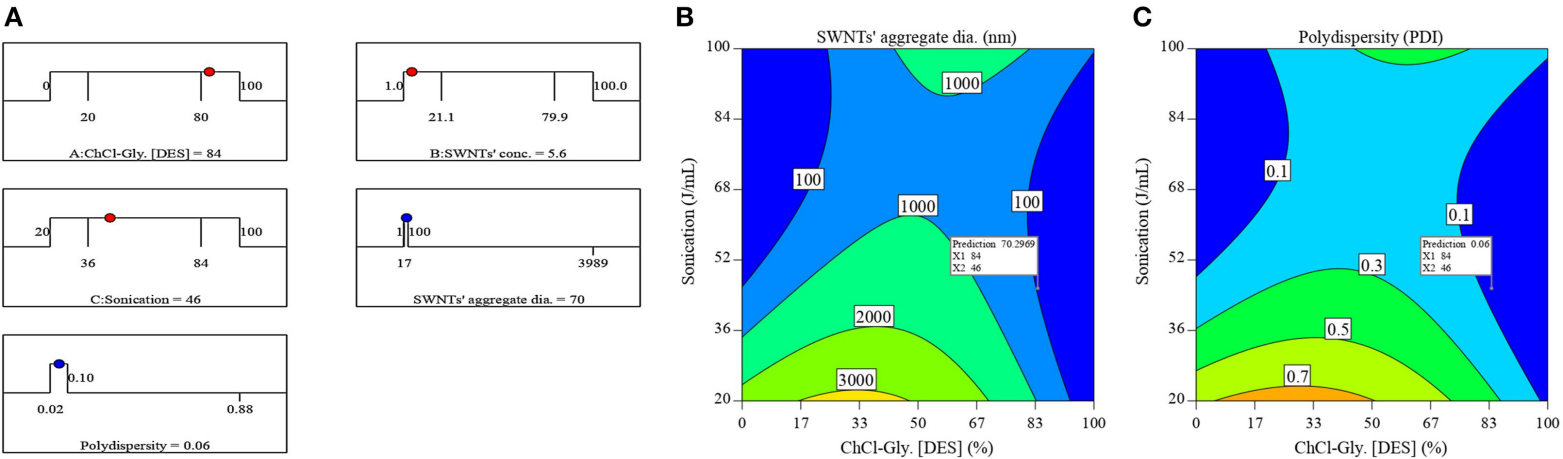
Optimization of SWNTs' dispersion in aqueous ChCh-Gly system. **(A)** The ramp plot indicates the position of factors (red dots) and responses (blue dots) at optimized positions. Optimum **(B)** SWNTs' aggregate diameter (70.3 nm) and **(C)** polydispersity (0.06) can be located by the position of flags.

The optimization was performed to obtain reasonably small SWNTs' aggregates with low polydispersity. The optimization criteria, ramp plots in Figure 6, were targeted at scanning the entire range of ChCl-Gly concentration in water (0–100%), SWNTs' concentration (1–100 mg/L), and sonication energy (20–100 J/mL) to obtain SWNTs aggregates ≤100 nm with polydispersity ≤0.1. The factors and responses are represented by blue and red dots, respectively. The optimization results are shown in Figure 6.

From Figure 6, SWNTs' dispersion in aqueous DES system could be optimized by processing 84% ChCl-Gly aqueous solution containing 5.6 mg/L SWNTs at 46 J/mL sonication energy. The optimum factors were expected to yield SWNTs' aggregates of 70 nm with 0.06 PDI as shown by ramps in Figure 6A and flags in Figures 6B,C. The experiment was performed, at optimized conditions, to verify this predictability of the response surface model. The SWNTs' dispersion, hence obtained, contained 64 nm average aggregate diameter and 0.08 PDI. These results are reasonably close to the predicted values (70 nm and 0.06 PDI) given the complex nature of SWNTs and aqueous DES system.

### Mechanism of Dispersion

**Figure 7:**
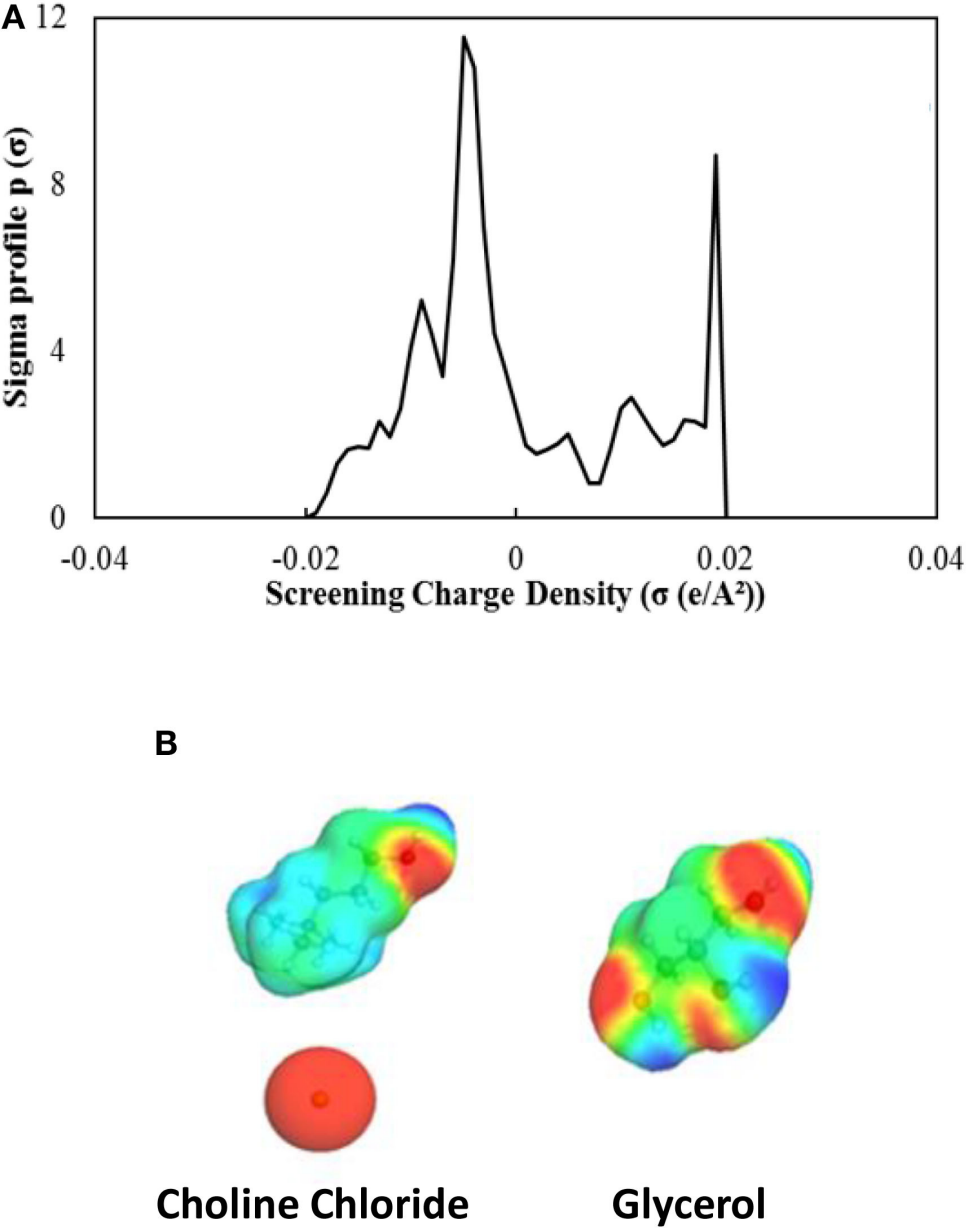
**(A)** The sigma profile and the **(B)** sigma surface (colored molecule diagrams) of the ChCl-Gly DES (calculated using COSMO-RS). The sigma surface represents the charge from negative (red) to positive (blue), where neutral is represented by green. The sigma profile, a weighted sum of all the profiles of all components, has two large peaks indicating both strong negative and positive screening charges in the mixture.

The aggregation and dispersion of the SWNTs are mainly influenced by the relative interplay between the inherent van der Waals attractive and the electrostatic repulsive interactions within the SWNTs. This phenomenon of aggregation and dispersion has been extensively reported in literature (Tucknott and Yaliraki, 2002; Tan and Resasco, 2005; Niyogi et al., 2007; Rajter et al., 2007; Saleh et al., 2008; Khan et al., 2013; Koh and Cheng, 2014; Zaib and Ahmad, 2019). The strong inter-tube van der Waals interactions cause SWNTs to re-aggregate after sonication, which is detrimental to the dispersion process. Consequently, there has been some devised techniques to alter the surface charge of the carbon nanotubes in their suspensions. Amongst the methods utilized include salt addition, polyelectrolytes, metal ion addition, and other surfactants (Zhao et al., 2005; Heister et al., 2010; Koh et al., 2011, 2012; Raja et al., 2018). However, the mechanism of dispersion of SWNTs in deep eutectic solvents is thought to be due to their ionic nature (without any need for surfactants and organic solvent dispersants). The ionic nature of the DESs would allow them to interact with the SWNTs which are negatively charged based on their zeta potentials (Polo-Luque et al., 2013; Vadahanambi et al., 2013). To observe the ionic character of the ChCl-Gly 1:2, Conductor-like Screening Model for Realistic Solvents (COSMO-RS) was used for determining the net surface charge on a molecular level (Figure 7) (Klamt, 1995). This approach is a known thermodynamic method for predicting chemical potentials (μ) in liquids using quantum chemistry (Rezaei Motlagh et al., 2019).

The COSMO-RS calculates the surface screening charge of the eutectic mixture, which can be visualized with the sigma surface (Figure 7B). The sigma surface, screening charge distribution, provides a qualitative estimation of the interaction energy based on the confined association of the polarization charge densities (Rezaei Motlagh et al., 2019). It could be observed that the DES showed significant negative surface charge (red) in addition to the positive charges (blue). This behavior signals the potentiality of the choline chloride and glycerol to form hydrogen bonds. As shown in Figure 7A, the sigma profile was obtained through the reduction of the screening charge density of the sigma surfaces of the molecules. It can be seen that the DES has an overall negative surface charge. A probable explanation might be the abundant prevalence of strong electronegative elements like chlorine and oxygen. The overall negative charge of the DES could be responsible for its electrostatic repulsive interactions toward the negatively charged SWNTs. These interactions would allow the stabilization of the SWNTs suspension in the DES and lessen their re-agglomeration tendencies. Similar findings of the electrostatic repulsion of molecular charges on SWNTs and their effect on the agglomeration has been reported by Polo-Luque et al. (2013) and Vadahanambi et al. (2013).

**Figure 8:**
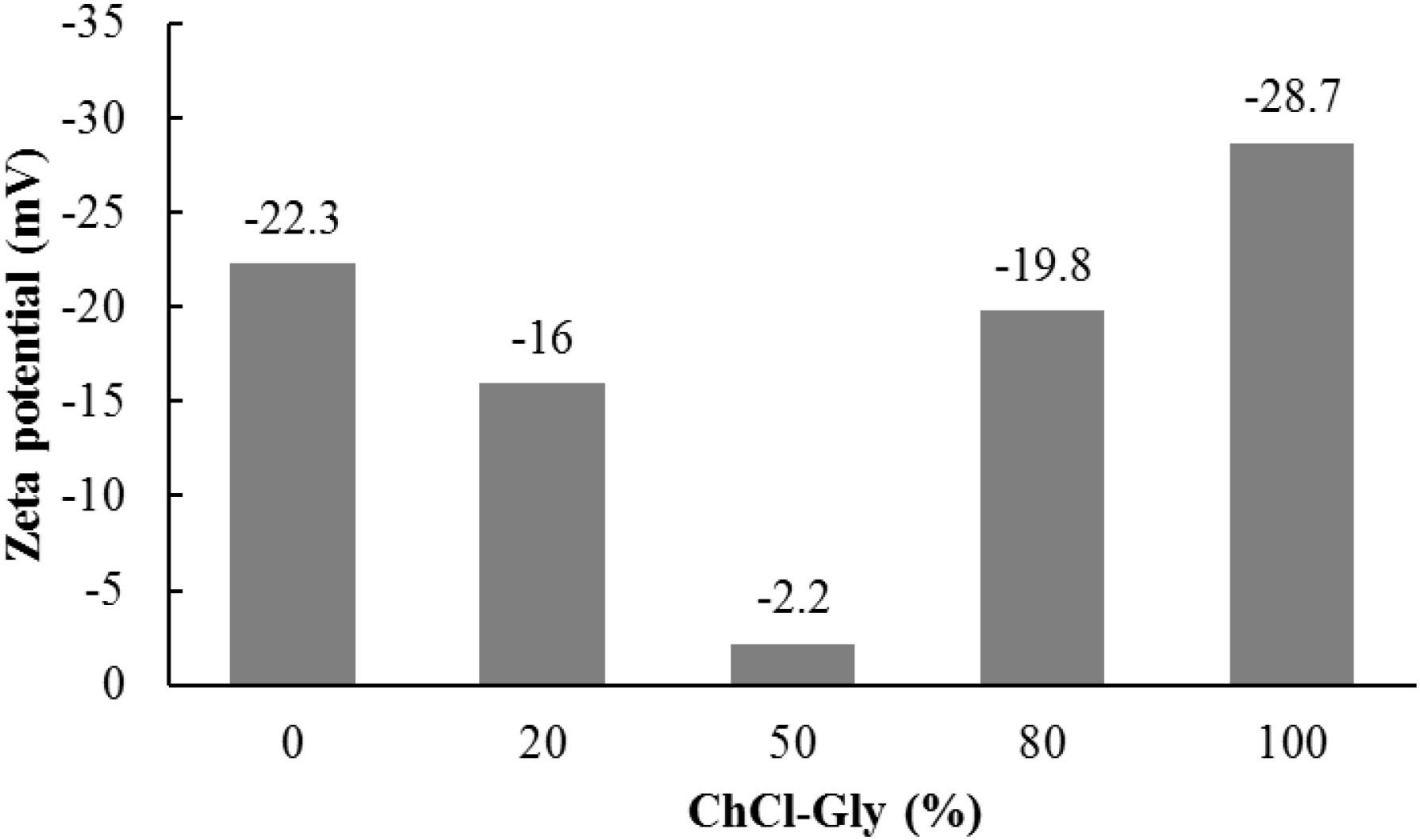
Zeta potential of SWNTs at various ChCh-Gly (mass% in water) concentrations. The negative surface charge of SWNTs decreased and then increased with the increase of ChCh-Gly (mass% in water). The surface charge of SWNTs almost neutralized (−2.2 mV) at 50% ChCh-Gly probably due to the disintegration of ChCh-Gly DES into its constituents.

The surface charge of SWNTs in aqueous ChCl-Gly system was determined at the various concentrations of ChCh-Gly (0, 20, 50, 80, and 100%) in water by measuring the electrical potentials at their edges, zeta potentials (Sun et al., 2008). Figure 8 shows the decrease and then increase in the magnitude of negative surface charge of SWNTs upon increasing ChCl-Gly concentration in water. In the absence of ChCl-Gly, the zeta potential of SWNTs was −22.3 mV which (negatively) decreased to −16 mV (at 20% ChCl-Gly) and further decreased to −2.2 mV (at 50% ChCl-Gly). However, more addition of ChCl-Gly does not continuously decrease the magnitude of negative surface charge, instead, it drastically increased (from −2.2 mV) to −19.8 mV at 80% ChCl-Gly concentration and finally reached at −28.7 mV in the presence of 100% ChCl-Gly DES. The colloidal dispersions having zeta potential |0–15| mV are considered unstable (Sun et al., 2008), therefore, zeta potential provides additional evidence for the stability of SWNTs at ChCl-Gly DES concentration of either below 20% and/or above 80%.

The neutralization of negative surface charge, in the presence of 50% DES, can be explained by the dissociation of ChCl-Gly into its respective salts in the presence of water as observed by Dai et al. (2015) (and discussed in the earlier section: Impact of Primary Factors on the Dispersion of SWNTs). The absence of H-bonding and a 100 times increase in ionic conductivity of the DES was observed upon adding 50–80% water in natural Choline Chloride based DESs (Dai et al., 2015). The increase in ionic conductivity is known to enhance aggregation kinetics of SWNTs by suppressing electrostatic repulsion via screening electrostatic charge on the surface of SWNTs (Saleh et al., 2010). Therefore, we speculate that the surface charge of SWNTs was neutralized due to the dissociation of DES into its respective salts at ~50% ChCl-Gly concentration in aqueous DES system. The absence of DES and high conductivity of background solution shielded the surface charge of SWNTs leading to the decrease of the zeta potential of SWNTs. The −2.2 mV zeta potential of SWNTs at 50% DES resulted in the aggregation of SWNTs which can be witnessed by the increase in average aggregate size and polydispersity of SWNTs in Figures 4, 5. However, a further increase in ChCl-Gly concentration (80–100%) improved the dispersion of SWNTs by reducing its aggregate size and polydispersity (Figures 4, 5) and increasing its negative surface charge to −28.7 mV. The increase in the negative surface charge of graphene by similar ChCl-Gly DES was observed by other researchers (Hayyan et al., 2015). The increase in the magnitude of negative surface charge of SWNTs, in the presence of 100% ChCl-Gly DES, can be attributed to the partial functionalization of SWNTs by the negatively charged molecules of ChCl-Gly DES (AlOmar et al., 2016, 2017).

### Practical Implications

In the literature, the studies aimed at improving the dispersion of SWNTs can be classified into either chemical modification of SWNTs surface (Hu et al., 2005; Di Crescenzo et al., 2009; Heister et al., 2010) or third component assisted dispersion (Ham et al., 2005; Pramanik et al., 2017). The chemical modification is usually accomplished through the use of corrosive acids, oxidizing agents, and/or other hazardous chemicals often leading to the permanent damage of the sidewalls of carbon nanotubes (Datsyuk et al., 2008). The third component assisted dispersion requires expensive solvents which are seldom cost-effective and rarely environmentally acceptable (Ausman et al., 2000; Amelio et al., 2014; Pramanik et al., 2017). This study utilizes comparatively inexpensive and environmentally benign deep eutectic solvent (DES) (Smith et al., 2014). The authors are unable to find any similar work where DES is systematically studied to disperse carbon nanotubes in water. Chen et al. dispersed multiwalled carbon nanotubes in ChCl-Gly-Urea DES in the presence of sodium dodecyl sulfate an anionic surfactant (Chen et al., 2019). However, their work cannot be compared due to the difference in materials (multiwalled vs. single walled carbon nanotubes), different DES (ChCl-Gly-Urea vs. ChCl-Gly), presence of surfactant, and absence of quantitative data (particle size, particle size distribution, dispersion quality, etc.) in their reported study.

## Conclusions

To summarize, the dispersion of SWNTs in deep eutectic solvent has been observed through experiments and RSM models. The RSM was successfully used to model and predict the dispersion of SWNTs. The results reveal complex dispersion behavior of SWNTs in aqueous system at various concentrations of ChCl-Gly, a commonly used deep eutectic solvent. The dispersion of SWNTs in DES was found to depend partially on the ChCl-Gly DES concentration in water, sonication energy, and concentration of SWNTs. It was observed that SWNTs tend to disperse uniformly in the presence of high (≥80%) concentrations of ChCl-Gly in water. The experimental conditions (ChCl-Gly concentrations in water, sonication energies, and SWNT concentrations) were optimized to obtain desired dispersions (small aggregates and low polydispersity) of SWNTs in aqueous DES systems. The SWNTs' aggregates of 64 nm and 0.08 PDI could be obtained by providing 46 J/mL sonication energy to a 5.6 mg/L SWNTs in 84% ChCl-Gly aqueous solution. The COSMO-RS modeling and zeta potential measurements helped in understanding dispersion mechanism. The negative surface charge of SWNTs altered from −22.8 mV (100% deionized water) to −2.2 (50% ChCl-Gly:deionized water) to −28.7 (100% ChCl-Gly). The non-uniform dispersion behavior of SWNTs in aqueous ChCl-Gly system was attributed to electrostatic interactions- originating from the disintegration of DES into its constituents at higher concentrations of water. This study can be helpful in estimating the dispersion of nanomaterials in aqueous DES systems for intended use.

## Data Availability Statement

All datasets presented in this study are included in the article.

## Author Contributions

QZ: conceptualization, methodology, investigation, validation, formal analysis, visualization, writing—original draft, and writing—review & editing. IA: formal analysis, visualization, and writing—review & editing. DW: validation, visualization, and writing—review & editing. IMA: supervision, validation, writing—review & editing, resources, project administration, and funding acquisition. All authors contributed to the article and approved the submitted version.

## Conflict of Interest

The authors declare that the research was conducted in the absence of any commercial or financial relationships that could be construed as a potential conflict of interest.
